# TRPC1 transcript variants, inefficient nonsense-mediated decay and low up-frameshift-1 in vascular smooth muscle cells

**DOI:** 10.1186/1471-2199-12-30

**Published:** 2011-07-12

**Authors:** Alexandra M Dedman, Yasser Majeed, Sarka Tumova, Fanning Zeng, Bhaskar Kumar, Christopher Munsch, Alan N Bateson, Jürgen Wittmann, Hans-Martin Jäck, Karen E Porter, David J Beech

**Affiliations:** 1 Multidisciplinary Cardiovascular Research Centre; 2 Institute of Membrane & Systems Biology, Faculty of Biological Sciences, Mount Preston Street, University of Leeds, Leeds, LS2 9JT, UK; 3 Yorkshire Heart Centre, General Infirmary at Leeds, Great George Street, Leeds, LS1 3EX, UK; 4 Division of Molecular Immunology, Nikolaus Fiebiger-Center for Molecular Medicine, University of Erlangen-Nürnberg, Glueckstrasse 6, D-91054 Erlangen, Germany; 5 Faculty of Medicine & Health, Clarendon Way, University of Leeds, Leeds, LS2 9JT, UK

**Keywords:** alternative splicing, nonsense-mediated decay, cationic channel, transient receptor potential canonical 1

## Abstract

**Background:**

Transient Receptor Potential Canonical 1 (TRPC1) is a widely-expressed mammalian cationic channel with functional effects that include stimulation of cardiovascular remodelling. The initial aim of this study was to investigate variation in TRPC1-encoding gene transcripts.

**Results:**

Extensive TRPC1 transcript alternative splicing was observed, with exons 2, 3 and 5-9 frequently omitted, leading to variants containing premature termination codons. Consistent with the predicted sensitivity of such variants to nonsense-mediated decay (NMD) the variants were increased by cycloheximide. However it was notable that control of the variants by NMD was prominent in human embryonic kidney 293 cells but not human vascular smooth muscle cells. The cellular difference was attributed in part to a critical protein in NMD, up-frameshift-1 (UPF1), which was found to have low abundance in the vascular cells. Rescue of UPF1 by expression of exogenous UPF1 was found to suppress vascular smooth muscle cell proliferation.

**Conclusions:**

The data suggest: (i) extensive NMD-sensitive transcripts of TRPC1; (ii) inefficient clearance of aberrant transcripts and enhanced proliferation of vascular smooth muscle cells in part because of low UPF1 expression.

## 1. Background

Most mammalian orthologues of the *Drosophila melanogaster *Transient Receptor Potential (TRP) channel are involved in regulated transmembrane Ca^2+ ^fluxes either because they are directly permeable to Ca^2+ ^or because they are permeable to Na^+ ^and therefore indirectly affect intracellular Ca^2+ ^[1,2]. TRPC1 was the first of the mammalian TRP channels to be cloned and has been found to be widely expressed throughout the body [3-5]. There is general agreement that it contributes to Ca^2+ ^and Na^+ ^entry but it should be appreciated that its functions often depend on heteromultimerisation with other TRP proteins or regulators [3,6]. TRPC1 and its associated TRPC channels are not voltage-gated ion channels but relatively slow chemically-modulated channels. Activation by depletion of Ca^2+ ^stores has been described but there is also stimulation by agonists of G protein-coupled receptors, oxidized phospholipids [3,7-9] and redox factors [10]. Important functions of TRPC1 have been indicated in many mammalian systems, including in cell hypertrophy, migration and proliferation [3,4,11]. In the cardiovascular system TRPC1 stimulates vascular smooth muscle cell (VSMC) hypertrophy and hyperplasia [12-14] as well as cardiac hypertrophy evoked by aortic constriction [11]. Furthermore, it is up-regulated in response to vascular injury [12] and metabolic syndrome [15] and down-regulated by exercise [15], consistent with it playing important roles in pathological cardiovascular remodelling.

Relatively little is known about the control of *TRPC1 *gene expression other than that there is regulation by NFκB, HIF-1 and Ca^2+ ^[16-18]. Splice variation of *TRPC1 *transcripts has been reported but there has been little investigation of the topic and so the extent and importance are unknown. One variant corresponded to 13 exons but other variants lacked one or both of exon 2 and exon 3, and thus contained only 11 or 12 exons [5,19,20]. Other variants, some with additional exonic sequences, have been suggested [21].

Nonsense-mediated decay (NMD) is a major RNA surveillance mechanism, degrading mRNAs that contain premature termination codons (PTCs) in eukaryotic cells [22-25]. Increasingly NMD is suggested to play roles in suppressing human diseases [22,26]. The first step in NMD involves attachment of an exon-junction complex 5' of exon-exon junctions during splicing in the nucleus. If mRNA lacks PTCs, exon-junction complexes are stripped during the first round of translation by the ribosome. However, the exon-junction complex recruits NMD factors if PTCs are detected at least 50 nucleotides upstream of the final exon-exon junction. Decapping and degradation of such transcripts then follows. A key NMD factor is the phosphoprotein up-frameshift-1 (UPF1, or RENT1). Decay of PTC-containing RNAs occurs when UPF1 interacts with UPF2 and UPF3 [23]. Although originally thought only to be a system for degrading aberrantly spliced transcripts, NMD and alternative splicing can couple together in a process termed regulated unproductive splicing and translation [27,28]. NMD has been suggested to be important in genetic cardiomyopathies [29] but, to the best of our knowledge, there is no information on the relevance to TRP channels, VSMCs, or vascular remodelling.

In this study we made a survey of splicing in human *TRPC1 *gene transcripts and investigated the potential relevance. The investigation primarily focused on proliferating human saphenous vein VSMCs obtained during coronary artery bypass graft surgery. Hyperplasia in these VSMCs is a key determinant of long-term failure of saphenous vein bypass grafts [12-14]. Human brain and aorta mRNA libraries and human embryonic kidney (HEK) 293 cells were used for comparison.

## 2. Results

### 2.1 Multiple *TRPC1 *transcripts containing premature termination codons (PTCs)

**Figure 1:**
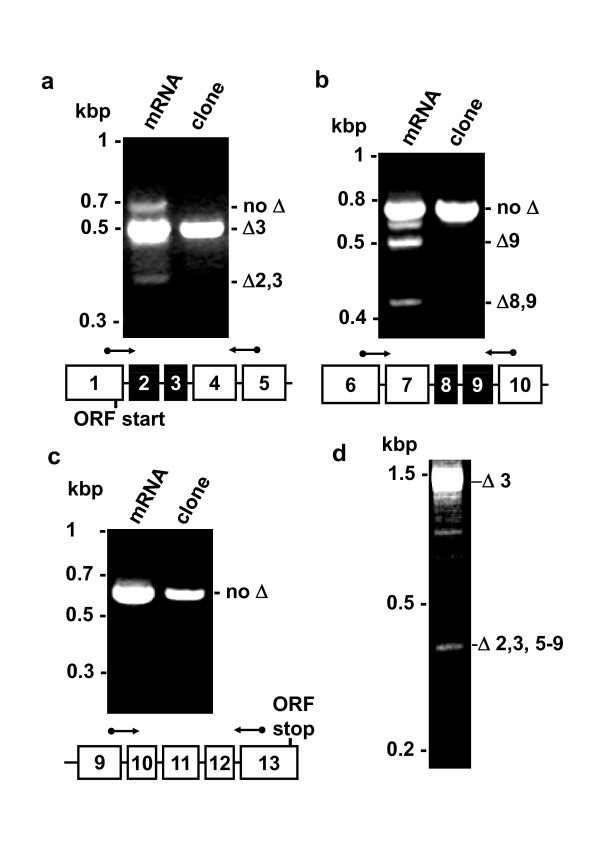
**Segmental RT-PCR scan of TRPC1 mRNA species**. (**a**-**c**) PCR on reverse-transcribed human brain mRNA ('mRNA') and the cloned human Δ3 TRPC1 cDNA ('clone'). As indicated by the exon schematics below each gel, PCR primers (forward and reverse arrows) spanned from exons 1 to 5 (**a**), 6 to 10 (**b**), or 9 to 13 (**c**). ORF: open reading frame. (**d**) Example raw data from PCR primers spanning from exon 1 to exon 10. Sequencing showed that the upper band was full-length sequence except for exon 3 (Δ3) and that the lower band lacked exons 2, 3 and 5-9 (Δ 2,3,5-9). (**a**-**d**) Experiments were repeated independently 4 times and yielded similar results.

A segmental RT-PCR scan was performed based on the predicted exonic structure of *TRPC1 *gene using human brain mRNA as a template. PCR primers spanning from exon 1 to 5 most noticeably amplified transcript lacking exon 3 (Figure 1a). A smaller product lacking exons 2 and 3 was also evident (Figure 1a). Primers spanning exon 6 to 10 revealed products lacking exons 8 and 9, or 9 alone (Figure 1b). In contrast, exon 9-13 PCR revealed only one full-length product, indicating absence of alternative splicing in this segment (Figure 1c). Parallel reactions performed in the absence of reverse transcriptase yielded no products, confirming that bands shown in Figure 1 arose from mRNA (the data are not shown but see below). For each primer pair, PCR was also performed using recombinant TRPC1 cDNA clone as a template control. The clone contained exons 1-2 and 4-13, and no intronic structure. PCR reactions on the clone revealed only single products, showing that the additional products from mRNA did not arise because of mis-priming on a single TRPC1 template (Figure 1a-c).

**Figure 2:**
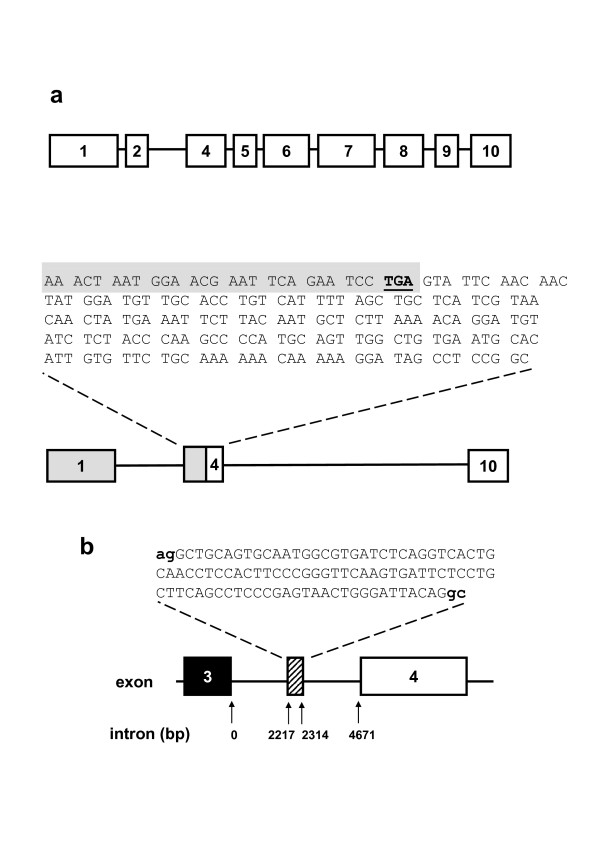
**Novel *TRPC1 *transcripts**. (**a**) Example TRPC1 splice variant in human brain: skipping of exons 2 and 3 causes a frame-shift and a PTC in exon 4 (bold, underlined). (**b**) Novel TRPC1 exon between exons 3 and 4 in human aorta. Exon-boundary donator and acceptor sites are marked.

**Figure 3:**
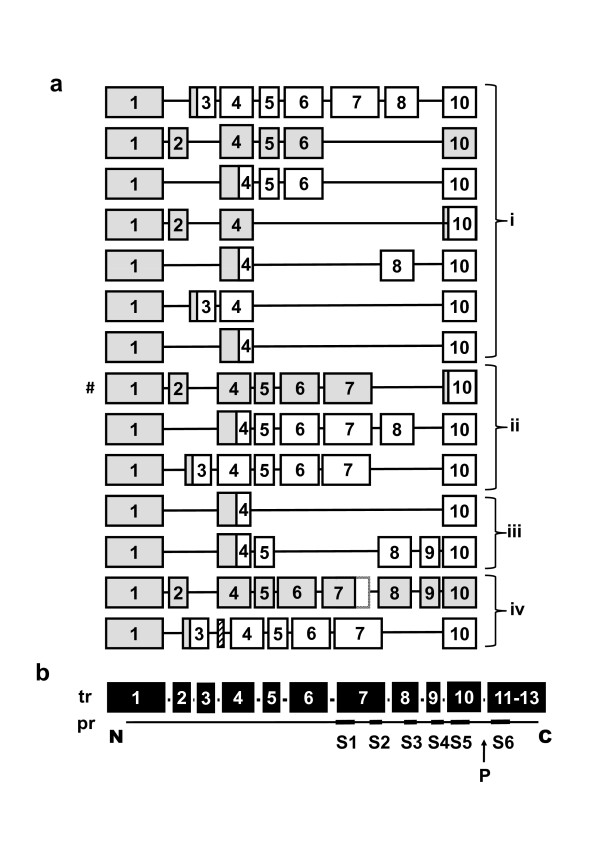
**Summary of sequenced *TRPC1 *transcripts**. (**a**) Results from long PCR and TOPO sub-cloning followed by direct DNA sequencing. Messenger RNA was from HEK 293 cells (i), human brain (ii), human saphenous vein (iii), and human aorta (iv). The numbers in the boxes indicate the exon numbers (5'-3', left to right). # indicates the Δ8,9 transcript studied in the functional assays of Figure 6. Grey shading indicates open reading frames (i.e. all but one shows a PTC). (**b**) Schematic showing the relationship between 13 primary exons of the transcript (tr) and the TRPC1 protein (pr) from amino (N) to carboxy (C) terminus and showing the transmembrane-spanning segments (S1-S6) and the ion pore region (P).

More detailed investigation was performed by using PCR primers spanning from exon 1 to exon 10 followed by sub-cloning and DNA sequencing of PCR products. Full-length transcript was evident but there were also smaller products. An example of the gel analysis is shown for human brain mRNA (Figure 1d). The approach was also applied to mRNA from HEK 293 cells, human saphenous vein, and human aorta. In each case, reactions of the type shown in Figure 1d were investigated by sub-cloning and sequencing to provide insight into the scope and characteristics of the splice variants. The experiments showed that multiple novel TRPC1 transcripts could be detected, including deletions of exons 2-3 and 5-9 (Figures 2 &3). Frame-shift and premature termination codon (PTC) insertion in exon 4 was observed to occur through deletion of exons 2 and 3 (Figure 2a). In one clone, a novel 98-bp exon (exon 3a) was observed with exon hallmarks ending in the CAG nucleotide sequence and having exon boundaries delineated by intronic AG acceptor and GC donor sites (Figure 2b). Although inclusion of exon 3a conferred a PTC it was made redundant by prior deletion of exon 2, resulting in a frame-shift in exon 3.

We did not attempt to define all splice variants or provide statistical analysis of the frequency of each variant but the data sample summarised in Figure 3a supports the conclusion that there is extensive splicing in *TRPC1 *transcripts. Out of the variants analysed, exons 2, 3 and 5-9 were omitted cassette exons whereas exon 4 was always present and thus constitutively spliced (Figure 3a). Transcripts contained frame-shifts that led to PTCs in or before exon 10 except in one case from human aorta where there was an in-frame deletion of 255 bp corresponding to the last half of exon 7 (Figure 3a). In Figure 3b the exons are aligned approximately with the six predicted transmembrane segments (S1-S6) and ion pore region (P) of the TRPC1 protein, showing that the insertion of PTCs will affect the transmembrane integrity and ion pore of the TRPC1 protein. Overall, the data suggest that PTC-containing variants are varied and complex, including in human blood vessels, and that they are likely to have negative effects on the generation and function of TRPC1 protein.

### 2.2 Cell type-dependent nonsense-mediated decay (NMD): low efficiency at *TRPC1 *transcripts in VSMCs

**Figure 4:**
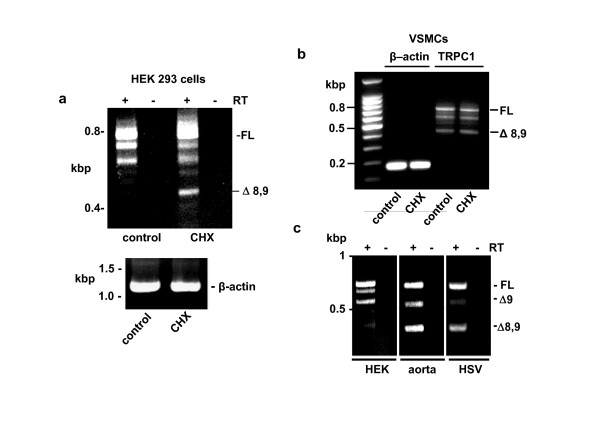
**Differential nonsense-mediated decay (NMD) of *TRPC1 *transcripts**. (**a**, **b**) In paired experiments, cells were pretreated with vehicle control (dimethylsulphoxide) or 25 μg/ml cycloheximide (CHX) for 6 hr prior to isolation of RNA and reverse transcription. PCR primer pairs spanned from exon 6 to 10 and data are shown with (+) and without (-) reverse transcriptase (RT) reaction. (**a**) HEK 293 cell data. The lower panel shows analysis of β-actin mRNA expression in the same samples. (**b**) Human saphenous vein VSMC data. (**c**) PCR with primers spanning exons 6 to 10. (**a**, **c**) Messenger RNA was analysed with (+) or without (-) reverse transcription (RT). (**a**-**c**) Experiments were repeated independently 4 times and yielded similar results.

Because NMD depends on first round translation it is acutely sensitive to cycloheximide, which inhibits peptidyl transferase activity of the 60S ribosomal subunit [30]. Therefore, to investigate the relevance of NMD, TRPC1 transcripts were investigated in cells after 6 hr treatment with cycloheximide or vehicle control using PCR primer pairs spanning from exon 6 to 10. In HEK 293 cells, cycloheximide increased the abundance of *TRPC1 *transcripts that had deletions of exons 8 and 9 (*TRPC1 *Δ8,9) (Figure 4a) or deletion of exons 2, 3 and 5-9 (data not shown). Cycloheximide had no effect on the abundance of the 'house-keeper' mRNA that encoded β-actin (Figure 4a). In contrast, in human saphenous vein VSMCs cycloheximide had no effect on *TRPC1 *transcripts (Figure 4b). Moreover, under basal conditions (i.e. in the absence of cycloheximide) the abundance of the *TRPC1 *Δ8,9 variant was apparently greater in VSMCs compared with HEK 293 cells (Figure 4b*cf *Figure 4a). This difference was also detected when comparing HEK 293 cell mRNA with mRNA from human aorta or human saphenous vein (Figure 4c).

Efforts were made to develop intra-exon PCR primers to enable real-time PCR quantification of exon expression. Unfortunately, because of the small size of the exons, we were only successful with intra-exon 4 primers, which target a constitutively spliced exon that was not different between samples (data not shown). We also note that the data of Figure 4a-c revealed PCR products in addition to those identified as Δ9 or Δ8,9 and which are unmarked in the figure panels. The sequences of these products were not determined. The abundance of these other PCR products may have decreased with increasing abundance of the Δ8,9 variant (Figure 4a) because they were out-competed in the multiple PCR reactions occurring within the single tube.

The data are consistent with the degradation of PTC-containing *TRPC1 *variants via the NMD pathway in HEK 293 cells but absence (or low efficiency) of such a mechanism in VSMCs.

### 2.3 Low UPF1 in VSMCs

**Figure 5:**
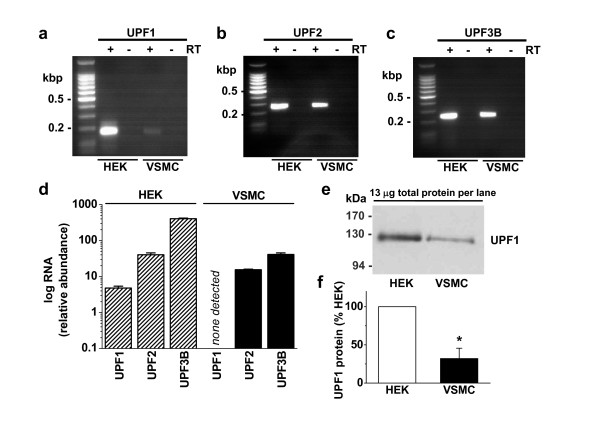
**Expression of UPFs 1-3**. (**a**-**c**) Example gels showing expression of mRNAs encoding UPF1 (**a**), UPF2 (**b**) and UPF3B (**c**) in HEK 293 and human saphenous vein VSMCs. Data are shown with (+) or without (-) reverse transcriptase (RT) reaction. (**d**) Mean data showing abundance of UPF1-3 mRNA species relative to β-actin in HEK 293 cells and VSMCs. (**e**) For a representative experiment, detection of UPF1 protein in HEK 293 cells and VSMCs. UPF1 was detected close to the expected molecular mass of 123 kDa. (**f**) Mean normalized data for experiments of the type illustrated in (**e**) (n = 3).

A possible explanation for the absence or inefficiency of NMD in the VSMCs is paucity of a key component of the NMD machinery such as UPF proteins. Analysis of mRNA species encoding up-frameshift (UPF) proteins revealed clear expression of UPF1, UPF2 and UPF3B in HEK 293 cells (Figure 5a-c). In contrast in VSMCs, mRNAs encoding UPF2 and UPF3B were readily detected but mRNA encoding UPF1 was difficult to detect (Figure 5a-c). The example experiment indicates a small amount of UPF1 mRNA (Figure 5a) but quantitative real-time PCR measurement was not possible because the mRNA species was undetectable in most samples (Figure 5d).

Protein abundance may be preserved by low protein turn-over in the face of low mRNA expression. Therefore, UPF1 protein was analysed by western blotting. Anti-UPF1 antibody labelled protein of the expected mass for UPF1, 123 kDa (Figure 5e). Two other anti-UPF1 antibodies labelled protein of the same size (data not shown). To determine UPF1 abundance, equal amounts of total protein were loaded from HEK 293 and VSMC lysates. Although UPF1 was evident in VSMCs, its abundance was 3.1 times less compared with HEK 293 cells (Figure 5e, f).

The data suggest that VSMCs have very low mRNA and relatively low protein abundance of UPF1, a critical component of the NMD mechanism.

### 2.4 Effect of UPF1 rescue on VSMC proliferation

**Figure 6:**
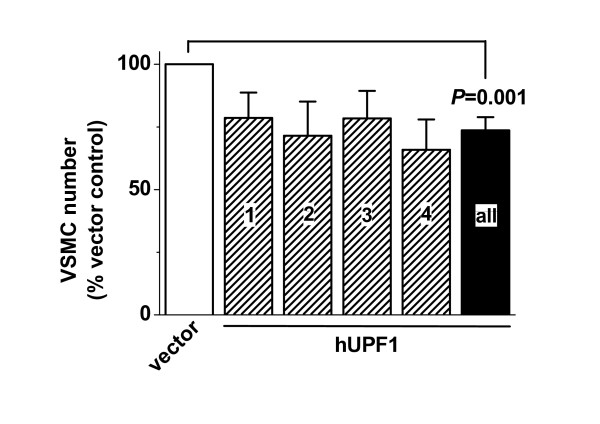
**Inhibition of VSMC proliferation by expression of exogenous hUPF1**. Mean normalised human saphenous vein VSMC numbers for 4 independent paired experiments on cells from 4 patients (labelled 1, 2, 3, 4), each comparing the effect of transfection with the vector and transfection with vector expressing human UPF1. Data from the 4 patients were combined (labelled 'all') for statistical analysis.

Previous studies have suggested that NMD is important as a suppressor of human diseases [22,26]. Coronary artery bypass grafts are carried out because individuals have coronary artery disease. The saphenous veins of these patients are prone to neointimal hyperplasia. Therefore, we hypothesised that a net effect of low UPF1 abundance may be to enhance proliferation of saphenous vein VSMCs. VSMCs were compared after transfection with control vector or vector expressing human UPF1 to rescue UPF1 levels. UPF1 suppressed VSMC proliferation (Figure 6).

The data suggest that low UPF1 and NMD in VSMCs stimulate cell proliferation and that the effect can be protected against by expression of exogenous UPF1.

### 2.5 Function of TRPC1 Δ8,9

**Figure 7:**
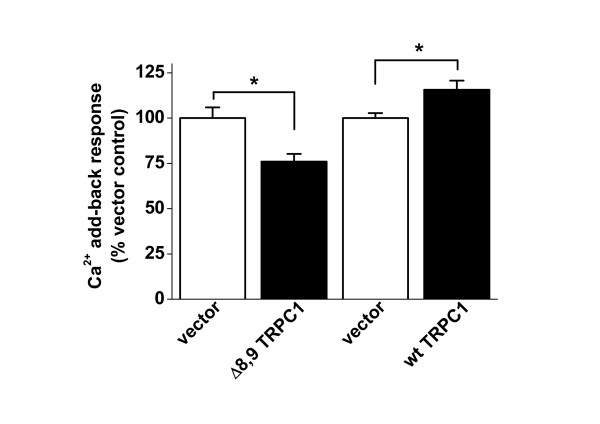
**Inhibition of Ca^2+ ^entry by over-expressed TRPC1 Δ8,9 in HEK 293 cells**. Measurement of the intracellular Ca^2+ ^concentration in transfected cells pretreated with 1 μM thapsigargin in zero Ca^2+ ^solution before addition of 1.5 mM Ca^2+ ^as indicated. Mean peak responses to 1.5 mM Ca^2+ ^are shown for paired experiments, one comparing vector (*n*/*N *= 11/39) with vector containing the TRPC1-Δ8,9 insert (*n*/*N *= 13/35), the other comparing vector (*n*/*N *= 3/60) with vector containing full-length (FL) TRPC1 without the Δ8,9 deletion (*n*/*N *= 3/45). Test data are normalised to their respective controls (vector only).

Protein encoded by the Δ8,9 deletion is predicted to comprise only the first two membrane-spanning segments of TRPC1 (see Figure 3b). The variant is susceptible to NMD in HEK 293 cells where it has low abundance (Figure 4a, c), but in VSMCs (where NMD is inefficient) there is significant mRNA encoding the variant (Figure 4b, c). To investigate if the variant has capability to be functional we generated it in a mammalian expression vector and transfected HEK 293 cells because these cells have low endogenous expression of the variant. Because TRPC1 is associated with Ca^2+ ^entry, the amplitude of Ca^2+^-entry was investigated in two paired sets of experiments: one comparing DNA vector with vector expressing the Δ8,9 variant; and the other comparing the DNA vector with vector expressing wild-type TRPC1. It was observed that the Δ8,9 variant partly inhibited Ca^2+ ^entry where as wild-type TRPC1 increased it (Figure 7).

The data suggest that short TRPC1 variants (such as Δ8,9) have an effect on Ca^2+^-entry that is opposite to that of wild-type TRPC1, causing inhibition.

## 3. Discussion

The data add to existing knowledge of alternative splicing in *TRPC1 *transcripts, showing that the splicing is extensive and commonly leads to frame-shifts with PTCs. These PTC variants are susceptible to NMD and may have no functional consequence unless NMD is compromised. Intriguingly, compromised NMD has previously been suggested in disease conditions and we observed weak or non-existent NMD in VSMCs from patients with coronary artery disease. Furthermore, we were able to show that weak NMD was associated with low expression of UPF1, a key protein of the NMD machinery. Therefore, although aberrant *TRPC1 *transcripts presumably reflect only a tiny fraction of the total aberrant variants of all genes in the cells, study of TRPC1 has led us to investigation of NMD and UPF1 in the vasculature and the first suggestion that they are relevant to unwanted vascular remodelling.

There is relatively little prior evidence that expression of UPF1 varies across cell types or changes in disease. Higher *Upf1 *mRNA abundance was detected in mouse testis but no differential expression was detected across a range of other physiological murine tissues [31]. Variation in expression of other NMD components occurred however, and up to 2-fold difference in NMD efficiency was suggested [31]. UPF1 is also a phosphoprotein and so its activity, rather than expression, may be a source of variation [23,24]. Stress factors such as reactive oxygen species may also inhibit NMD [32]. Nevertheless, as far as we are aware, our data provide the first evidence for markedly lower UPF1 mRNA and relatively low UPF1 protein in a cell or tissue type. Our data suggest that efficient NMD requires a certain abundance of UPF1 because the protein was not completely absent from VSMCs. It may be important that UPF1 was not completely absent because knock-out of the mouse gene (*Upf1*) is embryonically lethal [33] and strong knock-down of UPF1 arrests HELA cells in S phase [34]. It would, therefore, seem that mammalian cells require UPF1/Upf1. The origin and condition of the vein and VSMCs used in our experiments may have been influential because the cells of these veins will have been under stress in the patient and are particularly prone to proliferative and migratory behaviour, which is why there is a significant problem with subsequent neointimal hyperplasia [12,35]. Our data suggest that a previously unrecognised contributory factor in the aberrant proliferative behaviour may be compromised NMD due, at least in part, to low UPF1 expression. Consistent with this hypothesis, we were able to show that elevation (i.e. rescue) of UPF1 suppressed proliferation in VSMCs.

Short, N-terminal, variants of other TRP channel types have been described, including for TRPC2 [36], TRPC4 [37], TRPM1 [38] and TRPM2 [39]. Each variant is inhibitory for Ca^2+^-entry, as we describe for the Δ8,9 (N-terminal) variant of TRPC1. Therefore, it may be a common theme in TRP channels that there are inhibitory N-terminal variants that are variably expressed depending on activities of the spliceosome and NMD. However, it should be noted that although we showed native expression of Δ8,9 mRNA and other PTC-containing variants, we did not show that the variants led to proteins. Detection of endogenous ion channel proteins is notoriously difficult and the difficulty increases with splice variants that have even lower abundance. Few studies provide convincing evidence for native expression of such variants but instead show effects of heterologously over-expressed constructs. With a view to detecting endogenous variants we generated an anti-TRPC1 antibody targeted to amino acid sequence encoded by exon 1 (A Dedman & DJ Beech, unpublished data). Unfortunately, the antibody recognised over-expressed TRPC1 but lacked sufficient specificity to definitively identify short forms of endogenous TRPC1. Therefore, we cannot be sure that such short TRPC1 proteins exist in VSMCs even though we know the NMD mechanism is inefficient at *TRPC1 *transcripts and the mRNA species are present. If short TRPC1 proteins do exist endogenously they would be expected to suppress TRPC1-dependent Ca^2+ ^entry and consequently also VSMC proliferation [14]. This prediction may seem at odds with the observation that UPF1 suppressed cell proliferation (Figure 6). Nevertheless, it should be appreciated that consideration of UPF1 and NMD only in relation to TRPC1 would be unjustifiably restrictive; many aberrant splice variants are removed by NMD and it is the collective effect that will determine the net consequence on cell proliferation.

## Conclusions

The first half of this study identified extensive alternative splicing of TRPC1 transcripts that results in PTC-containing variants which are susceptible to NMD. These observations led us to find that NMD is weak or absent in proliferating VSMCs. Our data suggest that the weak NMD arises because of down-regulated expression of UPF1 and that rescue of UPF1 suppresses VSMC proliferation, a primary factor in the neointimal hyperplasia that leads to failure of veins as bypass grafts. We therefore suggest that inefficient NMD and down-regulated UPF1 are previously unrecognised features of human VSMC remodelling that result in unwanted transcripts with adverse effects. The findings support and expand previous suggestions that NMD plays a role in suppressing human diseases [22,26].

## 4. Methods

### 4.1 Saphenous vein and cell culture

Freshly discarded human saphenous vein segments were obtained anonymously and with informed consent from patients undergoing open heart surgery in the General Infirmary at Leeds. Approval was granted by the Leeds Teaching Hospitals Local Research Ethics Committee. The investigation conforms to principles outlined in the Declaration of Helsinki. Transfer of vein to the laboratory occurred in chilled Dulbecco's Modified Eagle's Medium (DMEM) and the processing of the vein for experiments occurred within 30-60 min after removal of the vein from the patient. VSMCs were prepared using an explant technique and grown in DMEM supplemented with 10% fetal calf serum (FCS), penicillin/streptomycin and L-glutamine at 37°C in a 5% CO_2 _incubator. Experiments were performed on cells passaged 2-5 times. Staining of cells positively for smooth muscle α-actin and smooth muscle-myosin heavy chain confirmed VSMC identity. HEK 293 cells were grown in Dulbecco's modified Eagle's medium-F12 media (Invitrogen) supplemented with 10% fetal bovine serum and penicillin (50 units/ml) and streptomycin (0.5 mg/ml) at 37°C in a 5% CO_2 _incubator. For cycloheximide (CHX) treatment, cells were grown to 60% confluency and treated with 25 μg/ml CHX or the vehicle control (dimethylsulphoxide) for 6 hr. Cells were washed with PBS before harvesting RNA.

### 4.2 RNA isolation and RT-PCR

Saphenous vein was placed in Hanks' solution (in mM: NaCl 137, KCl 5.4, CaCl_2 _0.01, NaH_2_PO_4 _0.34, K_2_HPO_4 _0.44, D-glucose 8, and HEPES 5), the medial layer dissected and snap-frozen immediately. Human brain total RNA and human aorta total RNA were purchased from Ambion (Huntingdon, UK). Messenger RNA was isolated using TRI-reagent (Sigma) and subjected to DNase I digestion (Ambion); 1-3 μg was reverse transcribed using oligo dT_(15) _or a *TRPC1 *gene specific primer with AMV-RT (Promega) or Transcriptor (Roche). Omitting reverse transcriptase controlled for the presence of genomic DNA. Thermal cycling was 95°C (5 min), 35 cycles (unless indicated) at 94°C (30 s), 55°C (45 s), and 72°C (2 min). PCR product (5 μl) was mixed with 1 μl loading buffer (Promega, UK) and resolved alongside appropriate DNA markers on 2% agarose-TAE gel containing ethidium bromide. Gels were deliberately loaded heavily with PCR product in order to reveal the lower abundance splice products. Therefore, high abundance products appear over-loaded. Bands were excised from gels and directly sequenced (Lark Technologies, Essex) or PCR products were subcloned using the TOPO TA system (Invitrogen) and resulting colonies sequenced using gene-specific or M13 primers.

### 4.3 Real-time quantitative PCR

Real-time RT-PCR cDNA was quantified using the Roche Lightcycler II system and LightCycler FastStart DNA Master SYBR Green I (Roche). The total RNA input into each reaction was constant. Total RNA abundance was quantified by Ribogreen assays (Invitrogen) using the fluorimeter function of the Lightcycler. DNA was amplified using the following protocol: hot-start at 95°C (10 min); 30 cycles of 95°C (10 s), 55°C (6 s) and 72°C (14-16 s). As previously described [40], PCR crossing points (C_p_) were determined using fit-points methodology (Light-cycler software 3.5) and RNA relative abundance calculated using 2^Cp (TRPC1)^/2^Cp (β-actin)^. C_p _values for β-actin were 15.7 ± 0.16 (HEK 293 cells) and 18.0 ± 0.13 (VSMCs). As appropriate, there was adherence to published guidelines for quantitative RT-PCR [41].

### 4.4 PCR primers

TRPC1 gene-specific RT primer (5'-3'): GTTAACCTGACTGTGTTGACAT. TRPC1 exon 1 forward primer: TCCATCCTCTTCCTCGC. TRPC1 exon 6 forward primer (5'-3'): ATTTAAGTCGTCTAAAACTTGCT. TRPC1 exon 5 reverse primer (5'-3'): AGCACTAAGTTCAAATGCT. TRPC1 exon 10 reverse primer (5'-3'): AGAAGAAACATCCCAAGAAAT. β-actin primers (5'-3'): ATGGATGATGATATCGCC (forward); CAAGAAAGGTGTAACGCAAC (reverse). TRPC1 intra exon 4 primers (5'-3'): AGCTGCTCATCGTAACA (forward); CCGGAGGCTATCCTTT (reverse). UPF1 primers (5'-3'): TTGACAGGATGCAGAGC (forward); GGCATAAACCTGGGAGT (reverse). UPF2 primers (5'-3'): AATGCTGATCGGGAGT (forward); TGTAGAATGCGCCTGT (reverse). UPF3B primers (5'-3'): CAACCTATGCCTGAGC (forward); ATCGATAGTCCCGACTT (reverse).

### 4.5 Western blotting

Confluent monolayers of VSMCs and HEK 293 cells were harvested in Lysis buffer (50 mM Tris, pH 8.0, 150 mM NaCl, 2 mM EGTA, 5% glycerol, 1% Triton X-100) containing protease inhibitors (Roche). For detection of UPF1, cell lysates were cleared by 5 min centrifugation at 8000 × g and protein concentration in the supernatants estimated by the BioRad DC protein assay. Equal amounts of protein (13 μg) were loaded and resolved by gel electrophoresis, transferred to nitrocellulose membrane, blocked with 5% milk in 150 mM NaCl, 20 mM Tris, pH 7.5, 0.1% Tween-20 and incubated (overnight at 4°C) with an antibody against human UPF1 (1:200) [42]. HRP-conjugated goat anti-rabbit secondary antibody (Sigma, 1:5000, 1 hr at room temperature) and SuperSignal PicoWest substrate (Pierce) were used for detection. Blots were also probed with an antibody against β-actin (Santa Cruz Biotechnology, 1:1000, overnight at 4°C) followed by HRP-conjugated bovine anti-mouse antibodies (Santa Cruz Biotechnology, 1:10,000, 1 hr at room temperature), but β-actin was found to be differentially expressed between HEK 293 cells and VSMCs. Normalisation of UPF1 abundance relative to β-actin yield a qualitatively similar result to that obtained by normalizing to total protein (these comparative data are not shown). Expression of UPF1 protein was also confirmed using anti-RENT1 antibody (Santa Cruz Biotechnology) and an antibody against human UPF1 (a gift from J. Lykke-Andersen, University of Colorado, USA).

### 4.6 Generation of Δ8,9 TRPC1

TRPC1-Δ8,9 (deletion of exons 8 and 9) was engineered by deletion PCR using as the template human TRPC1 (exon 3 deletion) in pIRES-EYFP (Clontech) and a primer pair designed to anneal to the 3' end of exon 7 and the 5' end of exon 10: TATTCTGTGGATTATTGATTTCAATGGGACAGATG (forward) and TCTGTCCCATTGAAATCAATAATCCACAGAATAAG (reverse). PCR conditions were 95°C (30 sec), then for 18 cycles, 95°C (30 sec), 55°C (1 min) and 68°C (16 min). The resulting construct was sequenced to confirm identity.

### 4.7 Ca^2+ ^imaging

Cells were transfected with hTRPC1-pIRES EYFP or hTRPC1Δ8,9-pIRES EYFP (3 μg) using lipofectamine 2000 (Invitrogen). Cells were split onto coverslips, and used for experiments 48-72 hr later. Cells were pre-incubated with 1 μM of fura PE3-AM (Calbiochem) at 37°C for 1 hr in standard bath solution (SBS, mM: NaCl 130, KCl 5, D-glucose 8, Hepes 10, MgCl_2 _1.2, CaCl_2 _1.5: pH 7.4 with NaOH), followed by 30 min wash period in SBS containing 1 μM thapsigargin. Recordings were made alternately from test and control cells. Fluorescence was observed with an inverted microscope (Zeiss, Germany), and a xenon arc lamp provided excitation light, the wavelength of which was selected by a monochromator (Till Photonics, Germany). Experiments were performed at room temperature and emission was collected via a 510-nm filter and sampled by a CCD camera (Orca ER; Hamamatsu, Japan). Images were sampled every 10 s at 345 and 380 nm and analysed off-line using regions of interest to select individual cells. [Ca^2+^]_i _is expressed as the ratio of the emission intensities for 345 and 380 nm (*R*_345/380_). Imaging was controlled by Openlab software (Image Processing &Vision Company Ltd, UK).

### 4.8 Cell proliferation and transfection with hUPF1 cDNA

VSMCs from human saphenous vein were transfected with cDNA encoding human UPF1 (accession number U59323.1) or control vector using the basic Amaxa protocol for primary smooth muscle cells (Lonza). Briefly, cells from confluent T75 flasks (passage 3-4) were released by detachin (Gelantis Inc. USA), pelleted and resuspended in Basic Nucleofector solution (Lonza). Equal numbers of cells from each patient were transfected with 2 μg/100 μl of hUPF1 in pCI-neo HA vector or empty vector using Amaxa electroporation system. Cells were plated onto 6-well plates with pre-warmed medium. After 2 days in DMEM supplemented with 10% FCS, cells were washed and harvested in detachin. Viable cells were identified by trypan blue exclusion and randomised samples were counted in a blinded fashion to assess proliferation. VSMC samples derived from 4 patients were used in the experiment and for each sample the effect on proliferation was determined from the ratio between number of cells that had been transfected with UPF1 and the control DNA.

### 4.9 Data analysis

Mean data are shown as mean ± s.e.mean. Statistical comparisons were made using Student's *t*-test between pairs of test and control data, where statistically significant difference is indicated by *P *< 0.05 (*). RT-PCR experiments were each repeated independently at least 4 times and yielded similar data; representative experiments are shown. For human vein experiments, similar results were achieved for at least 3 independent patient samples. For Ca^2+^-imaging, *n *is the number of independent experiments (coverslips) and *N *is the number of cells analysed per coverslip.

## List of abbreviations

TRPC1: Transient Receptor Potential Canonical 1; PTC: premature termination codon; NMD: nonsense-mediated decay; UPF1: up-frameshift-1; HEK: human embryonic kidney; VSMCs: vascular smooth muscle cells.

## Competing interests

The authors declare that they have no competing interests.

## Authors' contributions

All authors read and approved the final manuscript. AMD, YM, ST, FZ, ANB, DJB participated in research design. AMD, YM, ST, FZ, BK conducted experiments. CM, JW, HMJ, KEP contributed new reagents or analytical tools. AMD, YM, ST, FZ, DJB performed data analysis. AMD, YM, ST, JW, DJB wrote or contributed to the writing of the manuscript.

## Acknowledgements and funding

Supported by the Wellcome Trust and a University of Leeds PhD Studentship to YM.
